# The Vision of Cleanliness

**Published:** 1920-07-31

**Authors:** 


### The Vision of Cleanliness

### AND THE HEALTH FANATICS' NIGHTMARE.

Some very wise things were said with great vigour and in the exalted spirit of the true reformer by Mrs. George Cadbury, when she discussed the subject of personal and domestic hygiene before the members of the Royal Sanitary Institute. She rightly insisted on the observance of a proper sense of proportion in aiming at the sane mind in the sound body, and her remarks concerning the excesses of health fanatics are so good as to be worth giving in full:

"We are at the present time," she fearlessly declared, "outstripping the Greeks in an admiration of all that is strong and healthy and beautiful. A little more enthusiasm and we shall be in danger of ordering the destruction of the unfit infant at birth. Disease will be considered a crime. [As in Butler's *Erewhon*, she might have added.] We shall make it a penal offence to sit in a room or a railway carriage with closed windows. Individuals who sneeze in public and disseminate cold germs will be put in the stocks. Compulsory drill for all civilians will be conducted daily before breakfast in public squares; this will be followed by compulsory tooth drill and gargling. Our larder

**THE PUBLIC WELL-BEING**—(*continued*).

will be inspected weekly by a special band of women police. Accurate returns of the number of flies and mosquitoes destroyed by each child will be added to the school attendance charts."

Having directed this shaft of satire at the "fanatics," Mrs. Cadbury went to the root of the problem of promoting a better standard of health. It can only be accomplished by fostering and stimulating sustained effort. There are many signs that in many classes a higher conception of personal and domestic hygiene exists than there was a few years ago, and we believe that the newer teaching in the elementary schools has something to do with this improvement. But after all sustained effort is practically impossible in the slums. How can people, asks Mrs. Cadbury, living in a back-to-back house or a sunken alley possess any power of initiative or will to improve? Like so many other vital subjects of public health this question is linked up with the housing problem. From personal experience, extending now over twenty-five years, she knows what tremendous, almost incalculable, influence is exercised by favourable environment. She has watched the process of transformation in families who have moved out of congested areas into garden village homes. The great factor in the possibility of assimilating the education both of the mind and the body that the schools provide is through the multiplication of decent homes. Thus only can we become a nation of clean bodies; and a clean mind is the natural accompaniment of a clean body.

